# The Poly I:C maternal immune stimulation model shows unique patterns of brain metabolism, morphometry, and plasticity in female rats

**DOI:** 10.3389/fnbeh.2022.1022622

**Published:** 2023-01-06

**Authors:** Marta Casquero-Veiga, Nicolás Lamanna-Rama, Diego Romero-Miguel, Henar Rojas-Marquez, Julia Alcaide, Marc Beltran, Juan Nacher, Manuel Desco, Maria Luisa Soto-Montenegro

**Affiliations:** ^1^Laboratorio de Imagen Médica, Instituto de Investigación Sanitaria Gregorio Marañón, Madrid, Spain; ^2^Cardiovascular Imaging and Population Studies, Centro Nacional de Investigaciones Cardiovasculares (CNIC), Madrid, Spain; ^3^Departamento de Bioingeniería e Ingeniería Aeroespacial, Escuela Técnica Superior de Ingeniería, Universidad Carlos III de Madrid, Madrid, Spain; ^4^Department of Genetics, Physical Anthropology and Animal Physiology, University of the Basque Country (UPV/EHU), Leioa, Spain; ^5^Biocruces Bizkaia Health Research Institute, Barakaldo, Spain; ^6^Neurobiology Unit, Cell Biology Departament, BIOTECMED Institute, Universitat de València, Burjassot, Spain; ^7^CIBER de Salud Mental (CIBERSAM), Madrid, Spain; ^8^Fundación Investigación Hospital Clínico de Valencia, INCLIVA, Valencia, Spain; ^9^Advanced Imaging Unit, Centro Nacional de Investigaciones Cardiovasculares (CNIC), Madrid, Spain; ^10^Departamento de Bioingeniería e Ingeniería Aeroespacial, Universidad Carlos III de Madrid, Campus de Getafe, Madrid, Spain; ^11^High Performance Research Group in Physiopathology and Pharmacology of the Digestive System (NeuGut), University Rey Juan Carlos (URJC), Alcorcón, Spain

**Keywords:** mental disorders, schizophrenia, maternal immune stimulation, Poly I:C, sex differences, FDG-PET, MRI

## Abstract

**Introduction:** Prenatal infections are associated with an increased risk of the onset of schizophrenia. Rodent models of maternal immune stimulation (MIS) have been extensively used in preclinical studies. However, many of these studies only include males, omitting pathophysiological features unique to females. The aim of this study is to characterize the MIS model in female rats using positron emission tomography (PET), structural magnetic resonance imaging (MR), and neuroplasticiy studies.

**Methods:** In gestational day 15, Poly I:C (or Saline) was injected into pregnant Wistar rats to induce the MIS model. *Imaging studies*: [^18^F]-fluoro-2-deoxy-D-glucose-PET scans of female-offspring were acquired at post-natal day (PND) 35 and PND100. Furthermore, T2-MR brain images were acquired in adulthood. Differences in FDG uptake and morphometry between groups were assessed with SPM12 and Regions of Interest (ROI) analyses. *Ex vivo study*: The density of parvalbumin expressing interneurons (PV), perineuronal nets (PNN), and parvalbumin expressing interneurons surrounded by perineuronal nets (PV-PNN) were evaluated in the prelimbic cortex and basolateral amygdala using confocal microscopy. ROIs and neuroplasticity data were analyzed by 2-sample *T*-test and 2-way-ANOVA analyses, respectively.

**Results:** A significant increase in brain metabolism was found in all animals at adulthood compared to adolescence. MIS hardly modified brain glucose metabolism in females, highlighting a significant hypometabolism in the thalamus at adulthood. In addition, MIS induced gray matter (GM) enlargements in the pituitary, hippocampus, substantia nigra, and cingulate cortex, and GM shrinkages in some thalamic nuclei, cerebelar areas, and brainstem. Moreover, MIS induced white matter shrinkages in the cerebellum, brainstem and corpus callosum, along with cerebrospinal fluid enlargements in the lateral and 4th ventricles. Finally, MIS reduced the density of PV, PNN, and PV-PNN in the basolateral amygdala.

**Conclusion:** Our work showed *in vivo* the differential pattern of functional and morphometric affectation in the MIS model in females, as well as the deficits caused at the synaptic level according to sex. The differences obtained highlight the relevance of including both sexes in psychiatric research in order to consider their pathophysiological particularities and successfully extend the benefits obtained to the entire patient population.

## Introduction

Evidence suggests prenatal infections can result in chronic disorders that may manifest during adulthood. Thus, an environmental insult during pregnancy would be associated with an increased risk of the onset of mental disorders, such as schizophrenia and autism spectrum disorder (ASD). To study this complex interplay between prenatal infection and neurodevelopmental disorders, rodent models of maternal immune stimulation (MIS) have been extensively used in preclinical studies. In this sense, prenatal exposure to the immunostimulant polyinosinic: polycytidylic acid (Poly I:C), a synthetic agonist of the Toll-like receptor 3, induces maternal immune activation leading to a systemic inflammation that can affect the developing fetal brain (Hadar et al., 2015, 2018; Bikovsky et al., 2016; Careaga et al., 2017; Guma et al., 2021). Brain and behavioral deficits in offspring have been interpreted as relevant to a number of CNS disorders, including schizophrenia and ASD. But more importantly, the emerging consensus among researchers points out that prenatal immune challenge in combination with other environmental and genetic factors may lead to the onset of schizophrenia, ASD, or other CNS disorders (Careaga et al., 2017). Here we focus on schizophrenia although the prenatal immune challenge is relevant to a number of neurodevelopmental and neuropsychiatric disorders. Thus, the Poly I:C–MIS model reproduces the well-described delay in the occurrence of behavioral abnormalities in late adolescence (Hadar et al., 2015), and has shown high translational relevance to the three core domains of dysfunction in schizophrenia (i.e., positive, negative, and cognitive symptoms), as it elicits the behavioral, neurochemical, immunological, metabolic and structural changes related to this disorder (Hadar et al., 2015, 2018; Casquero-Veiga et al., 2019, 2021; Romero-Miguel et al., 2021), and has the optimal face, construct and predictive validities for schizophrenia (Jones et al., 2011).

Epidemiological and clinical studies have shown sex differences in patients with schizophrenia, indicating differences in neurodevelopmental processes, disease risk, and progression (Abel et al., 2010; Kraal et al., 2017; Adanty et al., 2022). In particular, the disease in men has an earlier onset compared to women, with a second peak of incidence in women coinciding with menopause (Ochoa et al., 2012). Indeed, it has been postulated that sex hormones may have an important role in the development of schizophrenia in females (Gogos et al., 2015). Moreover, the response to antipsychotic drugs is also different between sexes, with a better outcome in females compared to males (Abel et al., 2010; Kraal et al., 2017). Given the neurodevelopmental nature of the MIS animal model, and sex differences shown in this disorder, the study of both male and female offspring is a necessity in order to ensure greater translational value. In this respect, few studies have evaluated the particular deficits induced by MIS in each sex, mainly in behavior and immunohistochemical studies (Monte et al., 2017, 2020; Hui et al., 2018; Lins et al., 2019; Gogos et al., 2020), and none of them used functional exploratory *in vivo* brain imaging techniques, by means of Positron Emission Tomography (PET), as proposed in this study.

In humans, magnetic resonance studies have shown alterations in sex-specific neurodevelopmental and brain maturation trajectories, associated with mental disorders (Guma et al., 2017). Thus, larger ventricles, smaller temporal lobe, amygdala, and hippocampal volumes have been found in male patients with schizophrenia compared to female patients (Abel et al., 2010). Reduced hippocampal and amygdala volumes have also been described in first episode psychosis (FEP) patients (Pruessner et al., 2015) and individuals at clinical high risk (CHR) for psychosis (Guma et al., 2017), being more pronounced in male than in female patients. White matter tract asymmetries have also been found in fractional anisotropy between hemispheres in patients with schizophrenia (Bora et al., 2011; Steinmann et al., 2021) and in other components of the frontoparietal network (Miyata et al., 2012) with left hemispheric dominance in males and greater rightward assymetry in females (Steinmann et al., 2021). Moreover, PET studies have shown cortical and subcortical reductions in glucose metabolism at global and specific brain regions compared to controls (Townsend et al., 2022), but none of them has studied sex-differences. Some functional magnetic resonance imaging (fMRI) studies have also found sex differences in cerebral function in patients with schizophrenia and the first episode in brain areas associated with different cognitive domains (Mazza et al., 2021; Yang et al., 2021).

On the other hand, few *in vivo* imaging studies have been performed in the MIS model. Thus, adult MIS-offspring show enlarged ventricles and reduced hippocampus (Piontkewitz et al., 2011a; Casquero-Veiga et al., 2021; Romero-Miguel et al., 2021), together with metabolic reductions in the hippocampus and cortical regions, and metabolic increases in the nucleus accumbens, amygdala, and thalamus (Hadar et al., 2015; Casquero-Veiga et al., 2019). However, these studies have focused on male offspring and lacked gender comparison, omitting pathophysiological features unique to females. Therefore, in the present study, we sought to further characterize the schizophrenia-related effects of MIS, using several *in vivo* imaging modalities, in female offspring born to dams injected intravenously with Poly I:C during pregnancy. In the same cohort of Wistar rats, we evaluated the MIS effects on: (a) brain glucose metabolism at two neurodevelopmental stages; (b) brain morphometry on gray (GM), white matter (WM), and cerebrospinal fluid (CSF) in adulthood, and (c) brain neuroplasticity markers in the adult prefrontal cortex and amygdala.

## Material and Methods

**Figure 1:**
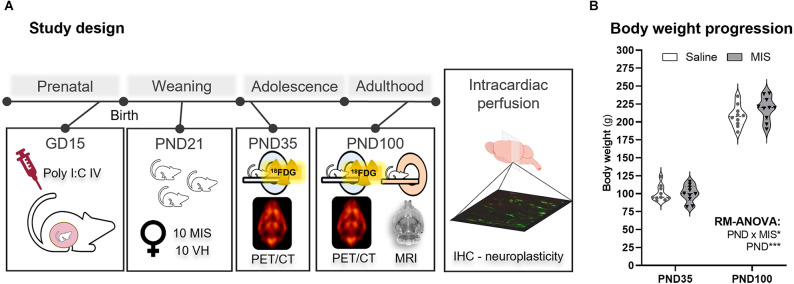
Study design and body weight changes in adolescence (PND35) and adulthood (PND100). **(A)** Schematic representation of the experimental procedures according to the age of the animals. **(B)** Violin plots showing body weight measurements at PND35 and PND100 for the Saline and maternal immune stimulation (MIS) groups. RM ANOVA followed by Bonferroni post-hoc test showed a clear statistically significant difference related to the Age factor (*p < 0.05, ***p < 0.001). The study factors included in this analysis were Age (PND, 35 and 100), and Phenotype (MIS, Saline, and MIS; *p* < 0.05).

A schematic representation of the study design is shown in Figure 1A. Supplementary Table 1 shows the MIS model reporting guidelines checklist.

### Maternal immune stimulation (MIS) model

On gestational day (GD) 15, 26 Wistar pregnant dams (12–15 weeks of age, 240–295 g) were injected with Poly I:C (storage at −20°C) diluted in saline (4 mg/kg, P0913, lot number 052M4035V, SIGMA, Germany), or saline solution (Saline) (0.18–0.24 ml of volume), through the tail vein, to induce the MIS model. Of note, sires matched in a 1:1 or 1:2 design, and female rats never been mated previously. Gestational time was evaluated by estrous cycle and formation of the vaginal plug. Poly I:C injections were performed under sevoflurane anesthesia (3% induction, 1.5% maintenance in 100% O_2_) at room temperature during the morning. Dams were housed individually after the immune challenge. Poly I:C-treated dams showed a body temperature about 0.5°C higher than Saline-treated dams at 2 h after the insult, normalizing to normal values at 24 h. On post-natal day (PND21), animals were weaned (ranging from 8 to 14 animals per litter: females ranging from 4 to 10, and males ranging from 2 to 8) and only 1–2 animals per sex from the same litter were included in the study. A randomization procedure was performed to assign the animals to each group. Two batches of animals were studied, one for imaging (females: N_MIS_ = 10 from seven litters, N_Saline_ = 10 from six litters), and the other for the neuroplasticity study (females: N_MIS_ = 8 from 12 litters, N_Saline_ = 8 from eight litters; males: N_MIS_ = 9 from 12 litters, N_Saline_ = 6 from eight litters). The experimental unit was a single animal. The animals were evaluated in a counterbalanced order including both experimental/control groups.

All animals were bred at the Animal Facility of Hospital Gregorio Marañón, maintained in conventional cages with wood shaving bedding type (Dates and), a temperature- and humidity-controlled vivarium, on a 12 h dark/light cycle (8:00 pm–8:00 am) with food (standard laboratory rat chow, 5LOS, Labdiet), and tap water available *ad libitum*. Cages were changed once per week. Males and females were housed in the same room. Social enrichment consisted of hiding structures. Animals were weighed immediately before each imaging acquisition protocol. Furthermore, animals were cytologically tested prior to euthanasia to ensure that all animals were in estrus. All experimental animal procedures were conducted according to European Communities Council Directive 2010/63/EU, following the ARRIVE guidelines (Percie du Sert et al., 2020) and approved by the Ethics Committee for Animal Experimentation of Hospital Gregorio Marañón.

### *In vivo* imaging acquisition

#### Positron emission tomography (PET)

PET studies were acquired in adolescence (PND 35) and adulthood (PND 100) with a small animal PET/CT scanner (ARGUS PET/CT, SEDECAL, Spain), under anesthesia with sevoflurane (3% induction, 1.5 maintenance in 100% O_2_). ~37 MBq of 2-deoxy-2-[^18^F] fluoro-D-glucose (FDG) were injected through the tail vein and, after an uptake period of 45 min, animals were scanned for 45 min. Reconstruction of PET images was performed using a 2D-OSEM algorithm, Full-Width Half Maximum (FWHM) of 1.45 mm, with a voxel size of 0.3875 × 0.3875 × 0.775 mm^3^ and an energy window of 400–700 keV. Decay and dead-time corrections were applied.

#### Computerized tomography (CT)

CT images were acquired to facilitate the co-registration of PET images for their analysis (Casquero-Veiga et al., 2021; Romero-Miguel et al., 2021). The same scanner mentioned above was used (340 mA, 40 kV, 360 projections, eight shots, and 200 μm of resolution) and images were reconstructed using an FDK algorithm (isotropic voxel size of 0.121 mm; Soto-Montenegro et al., 2014).

#### Magnetic resonance (MR)

MR images were acquired under sevoflurane anesthesia (3% induction, 1.5 maintenance in 100% O_2_) in adulthood (PND 100) with a 7-Tesla Biospec 70/20 scanner (Bruker, Germany). T2 spin-echo sequences were acquired, with TE = 33 ms and TR = 3,732 ms. The scan parameters were as follows: 70 slices measuring 0.4 mm in thickness, matrix size of 256 × 256 pixels, and FOV of 3.5 × 3.5 cm^2^. The artifact caused by the surface coil was corrected.

### Euthanasia and intracardiac perfusion

After completing the imaging protocols, animals were intracardially perfused under deep pentobarbital anesthesia, first with 0.9% Saline solution and then with 4% paraformaldehyde in sodium phosphate buffer (PB 0.1 M, pH 7.4). After perfusion, the brains were extracted from the skull and stored in PB 0.1 M. The two hemispheres were separated, and the left hemisphere was cut into 50 μm thick coronal sections with a vibratome (Leica VT 1000E, Leica Microsystems, Wetzlar, Germany). The sections were collected in 10 subseries and stored at 4°C in PB 0.1 M and sodium azide 0.05% until used.

### Immunohistochemistry

At adulthood, brain sections were washed in phosphate buffer saline (PBS) and then incubated for 1 h in 10% normal donkey serum (NDS; Abcys, Paris, France) in PBS with Triton X-100 (PBS-Tx; Sigma-Aldrich, San Luis, MO, USA). Afterwards, they were incubated for 48 h at 4°C with the primary reagents cocktail, diluted in PBS-Tx, and 5% NDS. The primary reagents cocktail consisted of guinea pig anti-PV antibody (1:9,500, Synaptic Systems, Gottingen, Germany), and *Wisteria floribunda* agglutinin (WFA; 1:710, Sigma-Aldrich, San Luis, MO, USA) for the detection of PNN (Härtig et al., 1992). After washing, sections were incubated for 2 h at room temperature with matching fluorescently labeled secondary reagents also diluted in PBS-Tx: A488-conjugated avidin (1:400, Invitrogen, Waltham, MA, USA) and CF555 donkey anti-guinea pig secondary antibody (1:400, Biotium, Fremont, CA, USA). Finally, sections were washed in PB 0.1 M, mounted on slides, and coverslipped using a fluorescence mounting medium (Dako North America Inc., Santa Clara, CA, USA).

### Study of the density of parvalbumin expressing cells, perineuronal nets, and parvalbumin expressing cells surrounded by perineuronal nets

Microphotographs of the prelimbic cortex (PrL) and basolateral amygdala (BLA) were obtained using a confocal microscope (Leica TCS SPE, Leica Microsystems, Wetzlar, Germany) with a 20× magnification objective. The resulting images were 550 μm × 550 μm single confocal planes per slice. A constant gain, exposure, and light intensity were used for all images and within regions. A total of three slices were photographed per animal and per region for the anterior, middle, and posterior parts of the PrL and BLA following the anteroposterior (AP) axis of the brain. The stereotaxic coordinates for the PrL were approximately +4.20 mm, +3.20 mm, and +2.20 mm anterior to Bregma, and the coordinates for the BLA were approximately −1.88 mm, −2.56 mm, and −3.14 mm posterior to Bregma.

### Data processing and statistical analysis

#### Body weight

Differences in total body weight between groups were evaluated by repeated-measures (RM) ANOVA followed by Bonferroni post-hoc analyses using GraphPad Prism 9 statistics software (GraphPad Software Inc., USA). The study factors included in this analysis were age (PND, 35 and 100) and phenotype (MIS, Saline, and MIS; *p* < 0.05). Outliers were evaluated using ROUT test (*Q* = 1%) and, if found, were removed from the analysis. Values are expressed as mean ± SEM.

#### PET analyses

PET images followed a pre-processing protocol previously described in (Soto-Montenegro et al., 2014). Briefly, PET scans were spatially co-registered (PETsreg) to a random reference CT scan (CTref; Pascau et al., 2009) normalized to the Paxinos and Watson rat brain atlas (Paxinos and Watson, 2008). Furthermore, an MRI scan was also registered to the same CT_ref_(MRI_reg_).

PET data were studied by voxel-by-voxel (SPM) and regions of interest (ROIs) analyses. The former followed a protocol previously described based on a data-driven normalization method (Gasull-Camós et al., 2017; Casquero-Veiga et al., 2021; Romero-Miguel et al., 2021). Thus, PET images were normalized in intensity to a non-significant area (NSA, PND35-NSA, PND100-NSA). NSAs comprised those brain areas that did not show statistically significant differences between groups after following an iterative method (Andersson, 1997), which was chosen in accordance with Shinohara et al.’s (2014) criteria. A whole brain mask segmented on the MRIreg was included in the analysis to eliminate voxels outside the brain. MIS and control groups were compared using 2-sample *T*-tests according to each age, using SPM12 software^1^. A significance threshold of *p* < 0.05 (uncorrected) was set and only significant regions of more than 50 activated connected voxels were accepted.

In addition, seven manual ROIs were segmented at each age (whole brain, prefrontal cortex, caudate-putamen, hippocampus, white matter, and pituitary gland) according to the Paxinos and Watson atlas (Paxinos and Watson, 2008). NSA masks were also included in the ROI analyses to rule out differences between groups. Whole brain and NSA data were evaluated by means of standardized uptake values (SUV), while the remaining ROIs data were normalized to their corresponding mean value of NSA at PND35 or PND100, respectively.

#### MRI analyses

MRI data were analyzed by voxel-based morphometry (VBM), as previously described in (Casquero-Veiga et al., 2019). The statistical analyses were performed using SPM12 and included the analysis of gray matter (GM), white matter (WM), and cerebrospinal fluid (CSF) in adulthood (PND100). Groups (MIS and Saline) were compared by 2-sample *T*-test analysis to obtain morphometry differences between them. Statistical and spatial significance thresholds were set at *p* < 0.05 (uncorrected) and k > 1,000 connected voxels, respectively. Only clusters that were statistically significant at the cluster level were considered.

Furthermore, manual ROIs of the whole brain, prefrontal cortex, caudate putamen, hippocampus, white matter, pituitary gland, and lateral ventricles were segmented on each MRI, following the brain coordinates of Paxinos and Watson atlas (Paxinos and Watson, 2008). Global brain volumetric changes were assessed using raw absolute data (i.e., cm^3^), while regional data for each brain structure were normalized to the global brain volume to obtain standardized percentages across subjects.

#### Immunohistochemistry

For image analysis, we used ImageJ (FIJI; Schindelin et al., 2012). PV+ cells, PNN, and their co-localization were quantified manually using the ImageJ multipoint function. Quantification and final densities for each brain region and counted structure were the averages of three images taken across the AP axis, as mentioned above.

The experimental design and statistical analysis were based on the indications of Diester et al. (2019). We first analyzed pooled data from both sexes and then data were segregated by sex and analyzed separately. However, we decided not to analyze sex as a between-subjects factor since the determination of differences between sexes was not an objective of our study. All slides were coded prior to quantitative analysis, and the code was not broken until the quantification was completed.

#### Statistical analysis of ROIs and immunohistochemistry data

Analysis was based on the number of animals used per group. Parametric methods were used. The normality and homocedasticity of each variable were tested using Shapiro-Wilk’s and Levene’s tests, respectively. Outliers were detected using the ROUT test (*Q* = 1%) and removed from analyses. SUV values of mean global FDG uptake were evaluated by RM-ANOVA, considering age (PND, 35 and 100) and phenotype (MIS, Saline, and MIS) as study factors (*p* < 0.05). Normalized PET ROIs, MRI ROIs and immunohistochemistry data were analyzed by 2-sample *T*-test analyses (*p* < 0.05). Probability values less than 0.05 (*p* < 0.05) were considered statistically significant. All the statistical analyses and graphs were obtained using GraphPad Prism 9 statistics software (GraphPad software Inc., USA). Values are expressed as mean ± SEM.

## Results

### Body weight is not directly affected by maternal immune stimulation in females

As expected, RM-ANOVA showed a clear statistically significant difference related to the Age factor (*p* < 0.001), as animals’ weight at PND35 was markedly lower than their body weight at PND100 (Figure 1B). Interestingly, although no differences were observed due to the MIS factor (*p* = 0.383), there was a statistically significant interaction between the factors (PND and MIS; *p* = 0.041). Nevertheless, both Saline (100.140 ± 3.402 g) and MIS (100.030 ± 3.707 g) animals showed very similar body weights at PND35, and this lack of differences was also evident at PND100 (Saline: 208.200 ± 4.638 g; MIS: 218.400 ± 5.256 g; Figure 1B).

### Brain glucose metabolism in females is hardly altered by MIS

#### Voxel-wise analyses

**Figure 2:**
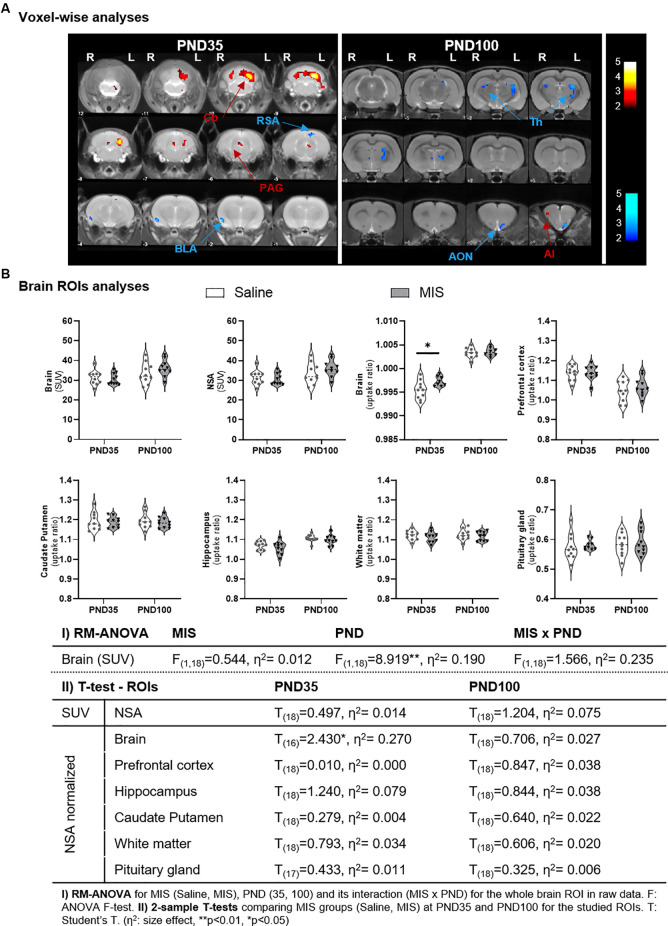
Differences in brain glucose metabolism at adolescence (PND35) and adulthood (PND100) evaluated by PET. **(A) Voxel-wise analyses**: colored PET overlays on the MR reference represent the *T*-maps resulting from the 2-sample *T*-test voxel-wise analyses between MIS vs. Saline comparisons at PND35 and PND100, indicating increased (hot colors) or decreased FDG uptake (cold colors). Color bars represent the *T*-values of the observed results in the left (L) and right (R) hemispheres. ROIs: AI, agranular insular cortex; AON, anterior olfactory nucleus; BLA, basolateral amygdala; Cb, cerebellum; PAG, periaqueductal gray matter; RSA, retrosplenial area; Th, thalamus. **(B)** **ROIs analyses**: Violin plots showing the global and regional metabolic values of MIS and Saline groups at PND35 and PND100 (*p < 0.05). Table shows MIS-related effects on brain metabolism in Saline and MIS offspring in adolescence and adulthood. Global raw values (mcps) were evaluated via RM-ANOVA (**p < 0.01). Regional analyses were performed on data normalized to a non-significant area (NSA) and were evaluated by 2-sample *T*-test analyses at PND35 and PND100 (*p < 0.05).

At PND35, MIS females showed a statistically significant reduction of FDG uptake in the basolateral amygdala (BLA) and retrosplenial area (RSA), along with a metabolic increase in the cerebellum (Cb) and periaqueductal gray matter (PAG), compared to the Saline group (Figure 2A, Table 1). However, this pattern is not maintained in adulthood (PND100), as females in the MIS group showed a statistically significant hypometabolism in certain regions of the thalamus (Th) and the anterior olfactory nucleus (AON), as well as hypermetabolism in the agranular insular cortex (AI), compared to the Saline group (Figure 2A, Table 1).

**Table 1:** Brain metabolic changes in maternal immune stimulation (MIS) vs. Saline, in adolescence and adulthood, in voxel-based analyses.

PET	ROI	Side	T	K	↓/↑	p_unc._ peak	p_FWE_ peak	p_unc._ cluster	p_FWE_ cluster
PND35	BLA	R	3.54	80	↓	0.001	0.745	0.454	0.991
	RSA	L	2.86	77	↓	0.005	0.971	0.463	0.992
	Cb	L	4.93	764	↑	<0.001	0.141	0.029	0.256
	PAG	R&L	2.20	53	↑	0.020	0.999	0.549	0.997
PND100	Th	L	3.23	225	↓	0.002	0.946	0.197	0.934
	Th	R	3.05	55	↓	0.003	0.976	0.529	0.999
	AON	L	2.36	61	↓	0.015	1.000	0.506	0.999
	AI	R	3.57	58	↑	0.001	0.833	0.517	0.999

*Side (left -L-, right -R-), *t*-value (T), type of change (↓ decrease, ↑ increase), *p*-value (*p*, uncorrected -unc.-, Family Wise Error -FWE-) at peak and cluster level, k (cluster size in voxels). ROIs: AI, agranular insular cortex; AON, anterior olfactory nucleus; BLA, basolateral amygdala; Cb, cerebellum; PAG, periaqueductal gray matter; PET, positron emission tomography; RSA, retrosplenial area; Th, thalamus*.

#### ROI analyses

RM-ANOVA of brain uptake showed a statistically significant effect of the PND factor (*p* = 0.0079), with a mean global uptake higher in adult animals (34.860 ± 1.078) than adolescent animals (30.999 ± 0.717), regardless of phenotype (Figure 2B). These differences lead us to compare groups separately according to their age, which required two normalization regions: one NSA for each age. Importantly, 2-sample *T*-tests showed no statistically significant differences in NSA uptake in either PND35 or PND100 between Saline and MIS animals. Similarly, no statistically significant differences were obtained in any other of the ROIs studied, with the exception of the mean brain uptake at PND35 (*p* = 0.03). At this time point, MIS females (0.997 ± 0.000) showed a statistically significant increase in FDG uptake compared to Saline animals (0.996 ± 0.001) (Figure 2B).

### Maternal immune stimulation induces several morphometric and volumetric changes in the brain of adult females

#### Voxel-wise analyses

**Figure 3:**
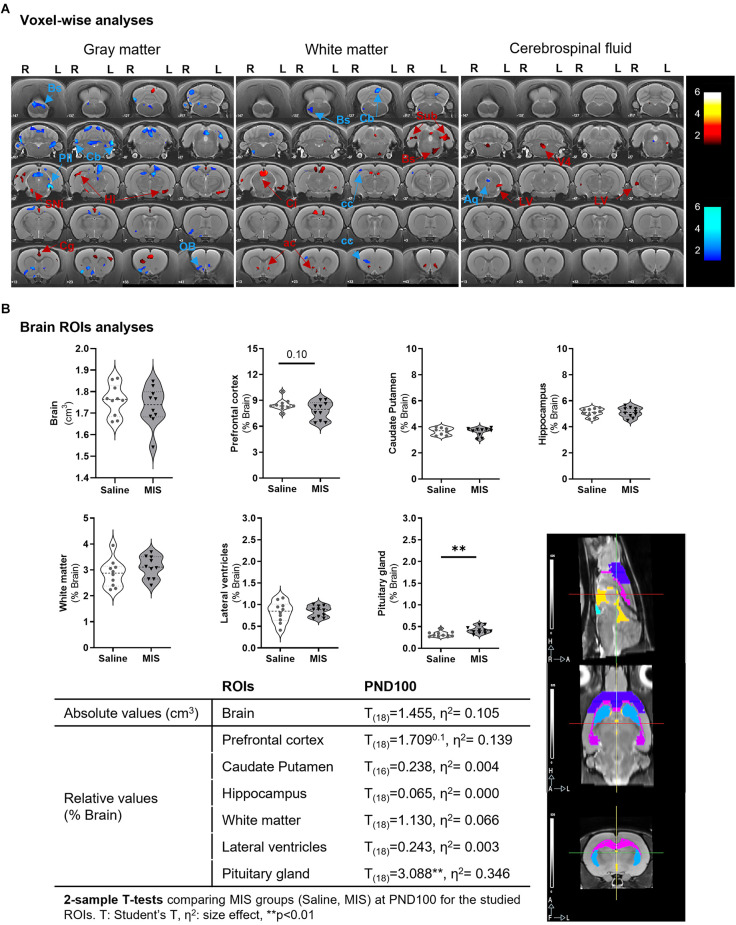
Morphometric and volumetric differences between Saline and MIS adult females evaluated by MRI. **(A) Voxel-based morphometry (VBM) analyses** in gray matter (left), white matter (middle), and cerebrospinal fluid (right). Results are shown in *T*-maps overlaid on a T2-MR template showing MIS phenotypic effect. *T*-values are represented by color bars showing enlargements (warm) or shrinkages (cold) in both left (L) and right (R) hemispheres. ROIs: AA, amygdalar hit; ac, anterior commisure; AQ, cerebral aqueduct; Bs, brainstem; Cb, cerebellum; cc, corpus callosum; Cg, cingulate cortex; Ci, colliculus; Hi, hippocampus; LV, lateral ventricle; OB, olfactory bulb; Pil, posterior intralaminar thalamic nucleus; Rs, retrosplenial cortex; Sin, substantia nigra; Sub, subiculum; V4, fourth ventricle. **(B) ROIs analyses**: Violin plots showing global and regional brain volume differences between MIS and Saline groups obtained *via* 2-sample *T*-test analyses (*p* = 0.10, ^**^p < 0.01). The table shows MIS-related volumetric differences between Saline and MIS offspring in adulthood. A graphical representation of the studied ROIs (prefrontal cortex -purple-, hippocampus -pink-, caudate putamen -blue-, lateral ventricles -yellow-, pituitary gland -aquamarine-) overlaid on a T2 MRI is shown in the lower right part of the figure.

VBM analyses showed different MIS-induced morphometric changes in GM, WM, and CSF in females (Figure 3A, Table 2). Thus, MIS caused a significant GM shrinkage in the posterior intralaminar thalamic nucleus, different cerebellar areas, olfactory bulb, and brainstem; together with a GM enlargement in the substantia nigra, cingulate cortex, and hippocampus. Simultaneously, these animals showed decreases in WM in the cerebellum, brainstem, and corpus callosum; as well as increases in WM in the colliculi, retrosplenial area, anterior commissure, amygdala area, subiculum, and brainstem. Finally, we also observed MIS-induced morphometric changes in CSF, showing shrinkage of the cerebral aqueduct and enlargements in the lateral and fourth ventricle as well as in the olfactory bulb.

**Table 2:** Morphometric differences in adult MIS vs. Saline females resulting from voxel-based morphometry (VBM) analyses of gray matter (GM), white matter (WM), and cerebrospinal fluid (CSF).

MRI	ROI	Side	T	K	↓/↑	p_unc._ Peak	p_FWE_ peak	p_unc._ Cluster	p_FWE_ cluster
GM	Pil	L	5.65	2,067	↓	<0.001	0.778	0.029	0.992
	Cb	L	4.75	6,106	↓	<0.001	0.997	0.001	0.108
	OB	L and R	4.60	1,503	↓	<0.001	0.999	<0.001	<0.001
	Cb	R	4.35	5,019	↓	<0.001	1.000	0.002	0.244
	Cb	L and R	4.34	2,5083	↓	<0.001	1.000	<0.001	<0.001
	Bs	L and R	3.75	3,489	↓	0.001	1.000	0.007	0.671
	Sin	R	3.36	1,650	↑	0.002	1.000	0.047	1.000
	Cg	L and R	3.28	7,159	↑	0.002	1.000	<0.001	0.049
	Hi	R	3.00	3,848	↑	0.004	1.000	0.005	0.547
	Hi	L	2.85	3,083	↑	0.006	1.000	0.010	0.809
WM	Cb	L	3.39	2,679	↓	0.002	1.000	0.001	0.179
	Bs	R	2.92	2,612	↓	0.005	1.000	0.001	0.201
	cc	R	2.53	1,634	↓	0.006	1.000	0.004	0.809
	cc	R	2.24	1,411	↓	0.012	1.000	0.007	0.936
	Ci	L and R	3.96	7,453	↑	0.001	1.000	<0.001	<0.001
	RS	L and R	3.58	2,069	↑	0.001	1.000	0.002	0.480
	ac	L	3.53	2,128	↑	0.001	1.000	0.002	0.440
	AA	L	3.37	2,009	↑	0.002	1.000	0.002	0.523
	Sub	L	3.29	3,736	↑	0.002	1.000	<0.001	0.030
	ac	R	3.01	2,401	↑	0.004	1.000	0.001	0.286
	Sub	R	2.97	2,577	↑	0.004	1.000	0.001	0.213
	Bs	L	2.42	3,269	↑	0.013	1.000	<0.001	0.065
CSF	AQ	L and R	2.70	1,263	↓	0.007	1.000	0.005	0.905
	LV	L	2.95	1,676	↑	0.004	1.000	0.002	0.546
	V4	L and R	2.86	2,983	↑	0.005	1.000	<0.001	0.039
	OB	L and R	2.75	3,760	↑	0.007	1.000	<0.001	0.008
	LV	R	2.43	1,259	↑	0.013	1.000	0.005	0.908

*Side (left -L-, right -R-), *t*-value (T), type of change (↓ decrease, ↑ increase), *p*-value (p, uncorrected -unc-, Family Wise Error -FWE-) at peak and cluster level, k (cluster size in voxels). ROIs: AA, amygdalar area; ac, anterior commisure; AQ, cerebral aqueduct; Bs, brainstem; Cb, cerebellum; cc, corpus callosum; Cg, cingulate cortex; Ci, colliculus; CSF, cerebrospinal fluid; GM, gray matter; Hi, hippocampus; LV, lateral ventricle; MRI, magnetic resonance imaging; OB, olfactory bulb; Pil, posterior intralaminar thalamic nucleus; Rs, retrosplenial cortex; Sin, substantia nigra; Sub, subiculum; V4, fourth ventricle; WM, white matter*.

#### ROI analyses

Despite the wide variety of changes observed in the voxel-wise analyses, ROI analyses only showed a statistically significant increase in the pituitary volume (*p* = 0.0063) in MIS (0.422 ± 0.026) vs. Saline (0.322 ± 0.020) females; and a non-significant reduction in prefrontal cortex volume (*p* = 0.105) in MIS (7.814 ± 0.332) vs. Saline (8.486 ± 0.211) (Figure 3B). No statistically significant differences were observed in any other ROI studied.

### Maternal immune stimulation with Poly I:C does not alter the density of parvalbumin expressing interneurons, perineuronal nets, or parvalbumin expressing interneurons surrounded by perineuronal nets in the prelimbic cortex

**Figure 4:**
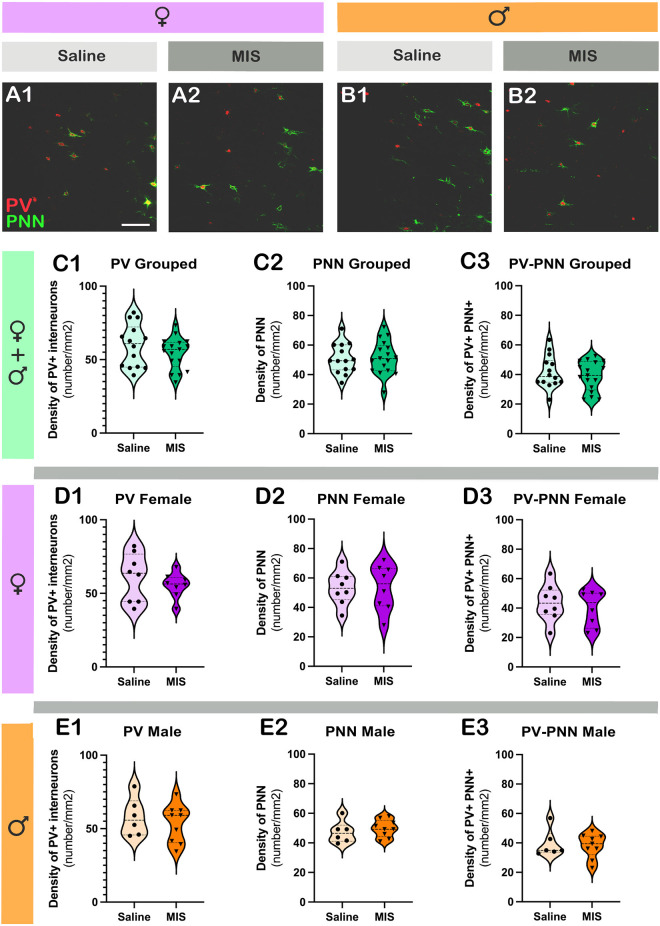
Analysis of the density of parvalbumin+ interneurons (PV) and perineuronal nets (PNN) in the prelimbic cortex, subregion of the mPFC. **(A,B)** Representative single confocal planes of parvalbumin+ interneurons (red, PV) and perineuronal nets (green, PNN) of control and MIS treated rats in females **(A)** and males **(B)**. **(C–E)** Graphs representing the effects of poly I:C on the density of PV + interneurons **(C1–E1)**, PNN **(C2–E2)**, and PV+ cells surrounded by PNN **(C3–E3)**. Graphs in the first row (green) include data from all experimental animals (not separated by sex; Saline: *n* = 14, MIS: *n* = 17). Graphs in the second row (purple) include data from females (Saline: *n* = 8, MIS: *n* = 8). Graphs in the third row (orange) include data from males (Saline: *n* = 6, MIS: *n* = 9). Values represent mean ± S.E.M. Scale bar 100 μm.

Figures 4A,B shows representative single confocal planes of parvalbumin+ interneurons (PV) and perineuronal nets (PNN) of control and MIS treated rats in females (A) and males (B). Table 3 shows the brain neuroplasticity changes in MIS vs. Saline, in females and males. When analyzing all animals together (without segregation by sex), the offspring of females injected with Poly I:C did not show significant differences when compared to the offspring of females injected with saline in the density of PV+ somata (Figure 4C1; *p* = 0.2681, *t* = 1.129) PNN (Figure 4C2; *p* = 0.8364, *t* = 0.2084) or of PV+ somata surrounded by PNN (Figure 4C3; *p* = 0.4558, *t* = 0.7560).

**Table 3:** Brain neuroplasticity changes in MIS vs. Saline, in females and males.

		mPFC			BLA		
		T	d	η^2^	T	d	η^2^
**Females + Males**	**PV**	1.129	29	0.042	3.753	20	0.413
	**PNN**	0.208	29	0.001	2.204	20	0.195
	**PV/PNN**	0.756	29	0.019	3.310	20	0.354
**Females**	**PV**	0.814	14	0.045	2.936	10	0.463
	**PNN**	0.025	14	0.001	2.384	10	0.362
	**PV/PNN**	0.594	14	0.024	2.714	10	0.424
**Males**	**PV**	0.649	13	0.031	2.201	8	0.378
	**PNN**	0.731	13	0.039	0.725	8	0.062
	**PV/PNN**	0.300	13	0.006	1.795	8	0.287

*Each column represents the *T* value, degrees of freedom (d) and Eta squared (η2) for data from all experimental animals, females and males in the medial prefrontal cortex (mPFC) and basolateral amygdala (BLA); (PV, parvalbumin+ cells; PNN, perineuronal nets; PV/PNN, PV+ cells surrounded by PNN)*.

In females, Poly I:C treatment did not induce changes in the density of PV+ somata (Figure 4D1; *p* = 0.4293, *t* = 0.8139), PNN (Figure 4D2; *p* = 0.9801, *t* = 0.02539) or PV+ somata surrounded by PNN (Figure 4D3; *p* = 0.5623, *t* = 0.5935). The same results were observed when analyzing males, in which there were no significant differences in the density of PV+ somata (Figure 4E1; *p* = 0.5277, *t* = 0.6489), PNN (Figure 4E2; *p* = 0.4780, *t* = 0.7306) or PV+ somata surrounded by PNN (Figure 4E3; *p* = 0.7689, *t* = 0.30000).

### Maternal immune stimulation with Poly I:C alters the density of parvalbumin expressing interneurons, perineuronal nets and parvalbumin expressing interneurons surrounded by perineuronal nets in the basolateral amygdala of females

**Figure 5:**
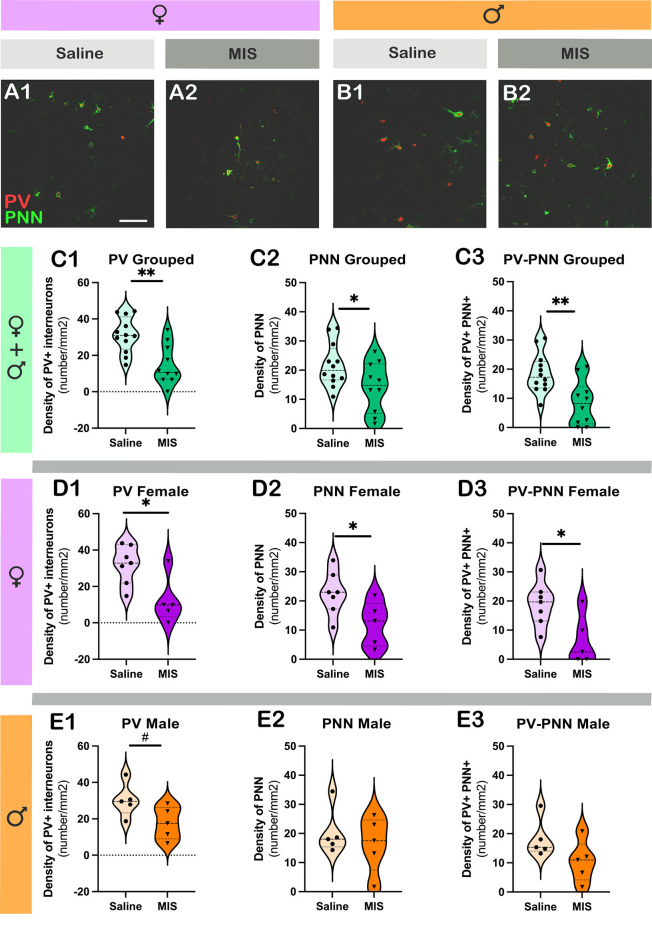
Analysis of the density of parvalbumin+ interneurons (PV) and perineuronal nets (PNN) in the basolateral amygdala. **(A,B)** Representative single confocal planes of parvalbumin+ interneurons (red, PV) and perineuronal nets (green, PNN) of control and MIS treated rats in females **(A)** and males **(B)**. **(C–E)** Graphs representing the effects of poly I:C on the density of PV + interneurons **(C1–E1)**, PNN **(C2–E2)**, and PV+ cells surrounded by PNN **(C3–E3)**. Graphs in the first row (green) include data from all experimental animals (not separated by sex) (Saline: *n* = 12, MIS: *n* = 10). Graphs in the second row (purple) include data from females (Saline: *n* = 7, MIS: *n* = 5). Graphs in the third row (orange) include data from males (Saline: *n* = 5, MIS: *n* = 5). Values represent mean ± S.E.M. Asterisks in graphs indicate statistically significant effects between groups (Saline x MIS) after unpaired Student’s *t*-test. Symbols: ^#^0.1 > *p* > 0.05; *p < 0.05; ^**^p < 0.01; Scale bar 100 μm.

Figures 5A,B shows representative single confocal planes of PV and PNN of control and MIS treated rats in females (A) and males (B). When considering both sexes together, in MIS treated animals there were significant decreases in the density of PV+ somata (Figure 5C1; *p* = 0.0013, *t* = 3.753), PNN (Figure 5C2; *p* = 0.0394, *t* = 2.204) and PV+ somata surrounded by PNN (Figure 5C3; *p* = 0.0035, *t* = 3.310). When considering only females, there was also a significant reduction in the density of PV+ somata (Figure 5D1; *p* = 0.0149, *t* = 2.936), PNN (Figure 5D2; *p* = 0.0384, *t* = 2.384) and PV+ somata surrounded by PNN (Figure 5D3; *p* = 0.0218, *t* = 2.714). When considering only males there was a tendency towards a reduction in the density of PV+ somata (Figure 5E1; *p* = 0.0589, *t* = 2.201), but neither the density of PNN (Figure 5E2) nor in that of PV+ somata surrounded by PNN (Figure 5E3) was altered (*p* = 0.4892 and *p* = 0.1104, respectively).

## Discussion

Sex-specific medicine has only begun to receive attention in the last few decades (Franceschini and Fattore, 2021). In particular, since the MIS model was proposed in the early 2000s (Zuckerman et al., 2003), few studies have evaluated sex differences in this model (Piontkewitz et al., 2012; Vorhees et al., 2015; Patrich et al., 2016; da Silveira et al., 2017; Drazanova et al., 2018), and even fewer if *in vivo* imaging studies are considered. Therefore, in this study, we have corroborated some of the schizophrenia-related changes occurring in female offspring of the Poly I:C MIS model by means of *in vivo* functional and structural imaging techniques, as well as *ex vivo* neuroplasticity studies. Here we discuss not only the potential implication of the observed deficits in the pathological phenotype but also the differences with the already published literature focusing on male offspring and patients.

First of all, MIS did not affect body weight during the development of female offspring. Therefore, despite the presence of a statistically significant interaction between study factors, this result was not supported by the *post-hoc* analyses. In fact, the actual differences between the two groups in each PND were insignificant, as the mean weights practically overlapped between groups. This lack of differences at different ages between Saline and MIS rats had been previously reported in male offspring by different authors (Bronson et al., 2011; Piontkewitz et al., 2011b; Yee et al., 2011). However, Bronson et al. (2011) found that female, but not male, offspring from MIS dams that underwent weight loss after Poly I:C injection, showed lower body weights than female offspring from Saline or MIS dams that did not suffer weight loss after Poly I:C insult. Unfortunately, weight differences in pregnant dams were not measured in this study in order to interfere as little as possible during gestation and up to delivery, which does not allow a comparison similar to that shown by Bronson et al. (2011). In any case, no further implication of these bodyweight differences in the MIS phenotype was reported.

Our results showed a significant effect of the PND factor in global brain metabolism, with higher values in PND100 vs. PND35, regardless of phenotype. These results are similar to that found by Guerrin et al. (2022), showing a progressive increase of brain metabolism with age in the frontal cortex in MIS male offspring. Besides, voxel-wise results showed increased FDG uptake in the cerebellum of MIS adolescent females compared to Saline animals, but this modulation was not present in adulthood. The cerebellar metabolic changes that we observed are relatively controversial. On the one hand, they mimic the hypermetabolism shown in males at PND21, which is not present in older ages (Guerrin et al., 2022) but highly contrast with the metabolic reduction found in males of the same age (i.e., PND35; Hadar et al., 2015). Therefore, the temporal nature of this metabolic feature could be indicative of the delayed neurodevelopmental impairment in females, implying a delay in achieving the same cerebellar metabolic reduction as observed in males. In fact, this effect coincides with the well-known delay in the age of symptom onset in women compared to men (Riecher-Rossler and Häfner, 2000; Hafner, 2003), which has also been well-ratified in the MIS-Poly I:C model (Piontkewitz et al., 2012). This delayed neurodevelopmental impairment may also explain the reduced thalamic metabolism observed in adult females of the MIS group, which again contrasts with the hypermetabolic pattern observed in males in early adolescence and adulthood (Hadar et al., 2015; Guerrin et al., 2022). In this respect, thalamic hypermetabolism has been associated with psychotic/positive symptoms in schizophrenia and related disorders (Silbersweig et al., 1995; Soyka et al., 2005), and their absence in females may be related to the later onset of these symptoms (Piontkewitz et al., 2012). However, the malfunction of specific nuclei present in the thalamus may result in their differential involvement in the cognitive, positive, and negative symptomatology of schizophrenia (Jiang et al., 2021). In this regard, dysfunction of the reticular nucleus of the thalamus has been associated with deficits in attentional processing and sensorimotor gating in patients with schizophrenia (Ferrarelli and Tononi, 2011). Furthermore, coinciding with our results in females, a metabolic decrease in this structure was observed in mice expressing a truncated form of the *Disrupted-in-Schizophrenia-1* (DISC1) gene (Dawson et al., 2015). The authors pointed to an alteration in GABAergic parvalbumin-positive neurons, which are highly abundant in this region, as a potential mechanism underlying the reticular hypofunction in this model (Dawson et al., 2015). Therefore, the hypometabolism shown in the thalamus in adult females might be a consequence of an early alteration of the cortico-thalamic-amygdalar pathway, which would have been evidenced by the early hypometabolism observed in both the retrosplenial cortex and basolateral amygdala in adolescent females. Thus, this reasoning would be supported by the consistent reduction in the density of PV expressing interneurons observed in the basolateral amygdala in both male and female rats of the MIS group (as discussed below). PV expressing interneurons are fast spiking cells that require a high level of energy and there is evidence for an important contribution of these cells to fMRI signal (Bucher et al., 2021). Then, our data support the concept that the thalamus is a central hub for projections within relevant structures involved in the pathophysiology of schizophrenia, leading to the appearance of different behavioral symptoms when these connections are disrupted (Steullet, 2020; Jiang et al., 2021). What it is still unclear is the timing of the onset of these deficits within the course of the pathology, as well as their involvement with the mechanisms underlying behavioral dysfunction in females.

Regarding volumetric results, although no differences in total brain volume were obtained in adulthood, a significant increase in pituitary gland volume was observed in the female MIS offspring compared to the Saline group. This hypertrophy is consistent with previous findings in adult male offspring (Casquero-Veiga et al., 2019) and in female first-episode patients (Pariante et al., 2004). In both cases, authors relate the pituitary volumetric abnormalities to the occurrence of hypothalamic-pituitary-axis disturbances and endocrine disorders in schizophrenia (Halbreich and Kahn, 2003; Pariante, 2008; Nordholm et al., 2013; Casquero-Veiga et al., 2019; Franceschini and Fattore, 2021). Interestingly, early treatment with risperidone prevented pituitary enlargement in the male adult offspring of this animal model (Abel et al., 2010). Therefore, our results in females support the previously suggested notion of pituitary gland volume as a biomarker of schizophrenia.

Likewise, lateral ventricular volume, as a marker of pathology progression and early antipsychotic treatment response in the Poly I:C MIS model, has been extensively validated in adult females and males, with this brain deficit being absent during adolescence (Piontkewitz et al., 2012; Casquero-Veiga et al., 2019). Accordingly, ventricular system enlargement at the expense of GM volume loss has also been reported in young subjects at high-risk for psychosis, as well as in patients with schizophrenia (Chung et al., 2017). Consistent with this background, our VBM results in adult female MIS offspring showed significant enlargement in the lateral and fourth ventricles, as well as a widespread reduction in several cortical regions, and an almost significant reduction in the prefrontal cortex, according to the ROI analysis. Therefore, given the proposed delay in the pathological course in females, it is conceivable that more conclusive changes would occur at later ages in female MIS offspring, thus reproducing the schizophrenia-related structural anomalies.

In addition, the VBM analysis of the WM showed shrinkage of the corpus callosum in adult MIS females vs. Saline controls. This is of particular interest considering the consistent decrease in WM volume and fractional anisotropy identified in patients with schizophrenia and bipolar disorder as shown in a recent meta-analysis, which included VBM and diffusion tensor imaging studies (Zhao et al., 2022). In contrast, inconclusive results have been obtained in the corpus callosum in the MIS model, showing increases in WM neuron density, particularly in females (Duchatel et al., 2016), but significantly smaller size, lower myelin/fiber structural development, and less effective fiber structure (Kreitz et al., 2020). In this regard, although the involvement of WM abnormalities in schizophrenia pathophysiology is clear, further studies are needed to unravel these WM deficits in the MIS model.

Another remarkable volumetric hallmark in the MIS model is the reduction in hippocampal volume, which has been proposed to mediate some of the cognitive deficits associated with this pathology (Piontkewitz et al., 2012; Patrich et al., 2016; Casquero-Veiga et al., 2019). In this regard, although MIS females showed a small cluster in the GM of the dorsal hippocampus suggesting a shrinkage of this structure, hippocampal enlargements were found in GM and WM, consistent with previous results from *ex vivo* MRI anatomical measurements involving an adult male and female Poly I:C mice (Kreitz et al., 2020). Thus, these contradictory results did not support the expected structural dysfunction at the hippocampal level associated with Poly I:C in adult females (Piontkewitz et al., 2012). These unexpected results likely respond to the neurodevelopmental delay that occurs in schizophrenia in females, which prevents us from detecting this hippocampal shrinkage and suggest the need to explore structural, behavioral, and metabolic deficits in MIS females at later time points compared to MIS males.

Regarding neuroplasticity results, we found that the density of PV-expressing interneurons, perineuronal nets (PNN), and PV-expressing interneurons surrounded by perineuronal nets (PV-PNN) in the basolateral amygdala were altered in the MIS model. Of importance, while these deficits were more pronounced in females, these neuroplasticity markers were also reduced in MIS males, although the differences did not reach statistical significance. Specifically, female MIS animals showed reduced density in these populations compared to control animals. In contrast to our results, quantitative and functional impairments in PV-positive cells, PNN, and PV-PNN have been previously demonstrated in the PFC of Poly I:C-MIS models in adulthood (Meyer et al., 2008; Canetta et al., 2016; Paylor et al., 2016), while the extent of these deficits varies markedly between studies. On the one hand, Meyer et al. (2008) already described a reduction in PV-positive cells in the prefrontal cortex and hippocampus of 6 month-mice prenatally exposed to Poly I:C. In fact, authors suggested that these deficits might have a potential implication for GABAergic systems in working memory abnormalities, particularly when the immune challenge occurred in late gestation, but could not draw a clear conclusion (Meyer et al., 2008). On the other hand, Paylor et al. (2016) evaluated PNN and PV-positive cells in the brain of the Poly I:C-MIS model in rats throughout the course of neurodevelopment, and found no changes in PV-positive cell density but a decrease in PNN and PV-PNN density in the PFC in early adulthood, and a reduction in PNN density in the amygdala in adolescence, supporting previous evidences of GABAergic dysfunction in the PFC of the Poly I:C-MIS model and patients with schizophrenia. Surprisingly, the amygdala deficits in PNN density observed by Paylor et al. (2016) were reversed in adulthood, which contrasts not only with our results but with previous studies in post-mortem brains of patients with schizophrenia (Pantazopoulos et al., 2010). As these authors pointed out, these neuroplasticity abnormalities reinforce the cortico-limbic impairment present in the Poly I:C-MIS model, which may underlie the deficits in fear processing described in Poly I:C offspring in adulthood (Pantazopoulos et al., 2010; Sangha et al., 2014). In any case, although the aforementioned studies include samples of both sexes, none of them have studied each sex separately.

Numerous studies have found an association between the dysregulation of PV expressing interneurons and excitatory/inhibitory (E/I) disturbances in psychiatric disorders (Nahar et al., 2021). Thus, disruption of this inhibitory control of cortical and subcortical circuits usually leads to glutamatergic and dopaminergic dysfunction that may in turn be responsible for schizophrenia symptomatology (Klimczak et al., 2021). In addition, another important regulator of interneuronal plasticity are the PNNs, which are part of the extracellular matrix located around the soma and dendrites of certain neuronal types, restricting their connectivity and plasticity (Carceller et al., 2020, 2022), Given their intimate relationship with PV expressing interneurons, PNNs play a key role in the activity of these interneurons and, therefore, in the E/I balance (Carceller et al., 2020, 2022). In this respect, our results would indicate the presence of an alteration of this E/I balance in the amygdala in MIS animals, suggesting the existence of poor neuronal maturation in this brain area.

Interestingly, sexual dimorphism has been observed in the developmental effects of PVs in different mental disorders, such as in the PFC in an early life stress model in mice (Goodwill et al., 2018) or in the striatum in an anxiety rat model (Ravenelle et al., 2014), and of PNNs in the amygdala in healthy mice (Ciccarelli et al., 2021). However, our study does not support sexual dimorphism in the development of PV expressing interneurons, PNNs, and PV-PNNs in the amygdala and prefrontal cortex in the MIS model.

Of note, the alteration of this E/I balance is accompanied by reduced brain metabolism in the amygdala in female animals in adolescence but increased metabolism in male animals (Hadar et al., 2015). Brain glucose metabolism measured by PET reflects predominantly the uptake of the FDG into astrocytes (Magistretti and Pellerin, 1999; Beard et al., 2022). Astrocytes are strongly activated after Poly I:C (Bernstein et al., 2016) and are in part responsible for the release of pro-inflammatory and anti-inflammatory cytokines found after MIS with Poly I:C (Ibi and Yamada, 2015). Of importance, a sex-specific neuroprotective and regenerative activity of glial cells have been found, which in turn may be responsible for the causes of sex differences in the pathological alterations of the nervous system (Chowen and Garcia-Segura, 2021). In addition, astrocytes have a functional role in glutamatergic neurotransmission, which is altered in schizophrenia (Tarasov et al., 2020). This may be associated with a hypofunction of NMDA receptors on PV-positive interneurons, resulting in decreased activity on PV-positive GABAergic interneurons, causing an imbalance in the E/I neurotransmitter actions (Notter, 2021). In addition, PV expressing interneurons require a continuous supply of oxygen and glucose through optimal mitochondrial functions (Kann, 2016), and their activity is susceptible to brain energy level changes (Pinna and Colasanti, 2021). Thus, if PV metabolic demands are lower, this may be related to reduced PV fast spiking activity or less dense excitatory innervation. Taken together, these different responses in glucose metabolism may be reflecting different strategies of male and female astrocytes to recover from the same insult.

Finally, we must acknowledge some limitations related to our work: First, the sample size prevents us from obtaining more robust results. Nonetheless, the number of animals in each group was found to be sufficient to detect significant changes in females and the same trend in males. Second, our work would have greatly benefited from the inclusion of behavioral studies as diagnostic tools for the evaluation of the MIS phenotype in females (Casquero-Veiga et al., 2021; Romero-Miguel et al., 2021). Third, given the developmental delay associated with females, exploring older ages in these animals would provide more accurate information on the expression of MIS phenotype. Fourth, only the left hemisphere was analyzed in the neuroplasticity study. Our PET and MRI results confirmed that many of our statistical differences are bilateral, and few of them are in one hemisphere, which would indicate a certain degree of asymmetry. Lastly, although voxel-wise analysis methods provide some correction for multiple comparisons, no further correction for multiple comparisons was applied. This decision was based on the voxel independence assumed by the Bonferroni correction, which is highly inaccurate given the spatial correlation between adjacent voxels (Lieberman and Cunningham, 2009; Verger et al., 2017). Nevertheless, spatial corrections were applied in order to prevent type I errors.

## Conclusions

Preclinical studies in the neuropsychiatric field using the MIS model lack female inclusion. This is of particular relevance given that gender differences have been found in the course of the disease or treatment response in patients with schizophrenia. In this sense, although there are some commonalities, we have found differences in the neurodevelopment of male and female offspring of the MIS model in brain metabolism, morphometry, and neuronal plasticity. Thus, it remains an open question whether there really is a delay in the MIS phenotype in females or whether it is just a matter of differential manifestation of the model between the sexes. In any case, our results highlight the need not only to include female animals in preclinical studies but also to develop sex-specific preventive and treatment strategies according to this sex-neurodevelopmental delay of the disease.

## Data Availability Statement

The original contributions presented in the study are included in the article/Supplementary material, further inquiries can be directed to the corresponding author/s.

## Ethics Statement

The animal study was reviewed and approved by Ethics Committee for Animal Experimentation of Hospital Gregorio Marañón.

## Author Contributions

MC-V: animal handle, analysis of imaging studies, writing—original draft, review and editing. NL-R and DR-M: animal handle, review and editing. HR-M: analysis of imaging studies. JA and MB: performance and quantification of biochemical studies. JN and MD: review and editing. MS-M: conception of the study, acquisition and analysis of imaging studies, animal handle, writing—original draft, review and editing. All authors contributed to the article and approved the submitted version.

## Footnotes

^1^ http://www.fil.ion.ucl.ac.uk/spm/software/spm12/
