# Supplementary material for: The Poly I:C maternal immune stimulation model shows unique patterns of brain metabolism, morphometry, and plasticity in female rats
Source: Front Behav Neurosci. 2023 Jan 6;16:1022622. doi: 10.3389/fnbeh.2022.1022622 (PMC9888250; doi:10.3389/fnbeh.2022.1022622)
Supplement: Supplementary file 1 [file Table_1.docx]

| **MIA model reporting guidelines checklist** | | |  |
| --- | --- | --- | --- |
| **ARRIVE Reporting Guideline & Recommendation** | **Arrive item** | **MIA Model Specific Reporting Recommendation** | **Page** |
| **Study design** | **6** |  |  |
|  |  | Number of experimental and control groups. | 5 |
|  |  | Randomization procedure to allocate animals to each group | 5 |
|  |  | Experimental unit | 5 |
| **Experimental procedure** | **7** |  |  |
|  |  | Compounds: source, vehicle, preparation/storage, administration route, volume administered, whether anesthetics were used at time of immune challenge. | 5 |
|  |  | Housing variables at injection | 5 |
| **Experimental animals** | **8** |  |  |
|  |  | Species, sex, strain, developmental stage | 5 & 6 |
|  |  | Maternal/offspring physiological variables | 5 |
| **Housing and husbandry** | **9** |  |  |
|  |  | Caging systems, animal holding room, bedding, social and physical enrichment | 5 |
| **Sample size** | **10** |  |  |
|  |  | Maternal and offspring N, weight, Sex distribution | 5 & 9 |
| **Allocating animals to experimental groups** | **11** |  |  |
|  |  | How many offspring per litter were used in each measure | 5 |
|  |  | Randomization/Matching procedures | 5 |
| **Experimental outcomes** | **12** |  |  |
|  |  | Ages of offspring at imaging and neuroplasticity evaluation | 5 & 6 |
|  |  | Order of testing | 5 |
| **Statistical disclosures** | **13** | Unit of analysis | 8 |
